# The hemodynamic initial-dip consists of both volumetric and oxymetric changes reflecting localized spiking activity

**DOI:** 10.3389/fnins.2023.1170401

**Published:** 2023-05-25

**Authors:** Ali Danish Zaidi, Niels Birbaumer, Eberhard Fetz, Nikos Logothetis, Ranganatha Sitaram

**Affiliations:** ^1^Department of Physiology of Cognitive Processes, Max Planck Institute for Biological Cybernetics, Tübingen, Germany; ^2^Institute for Medical Psychology and Behavioral Neurobiology, University of Tübingen, Tübingen, Germany; ^3^Department of Physiology and Biophysics, University of Washington, Seattle, WA, United States; ^4^Center for Imaging Sciences, Biomedical Imaging Institute, University of Manchester, Manchester, United Kingdom; ^5^Institute of Biological and Medical Engineering, Pontificia Universidad Católica de Chile, Santiago, Chile; ^6^Department of Psychiatry and Section of Neuroscience, Pontificia Universidad Católica de Chile, Santiago, Chile; ^7^Multimodal Functional Brain Imaging and Neurorehabilitation Hub, Diagnostic Imaging Department, St. Jude Children's Research Hospital, Memphis, TN, United States

**Keywords:** functional neuroimaging, near infra-red spectroscopy, neurovascular coupling, electrophysiology, visual cortex, primate, hemodynamics, initial dip reflects spiking activity

## Abstract

The initial-dip is a transient decrease frequently observed in functional neuroimaging signals, immediately after stimulus onset, believed to originate from a rise in deoxy-hemoglobin (HbR) caused by local neural activity. It has been shown to be more spatially specific than the hemodynamic response, and is believed to represent focal neuronal activity. However, despite being observed in various neuroimaging modalities (such as fMRI, fNIRS, etc), its origins are disputed, and its precise neuronal correlates are unknown. Here we show that the initial-dip is dominated by a decrease in total-hemoglobin (HbT). We also find a biphasic response in deoxy-Hb (HbR), with an early decrease and later rebound. Both the HbT-dip and HbR-rebound were strongly correlated to highly localized spiking activity. However, HbT decreases were always large enough to counter the spiking-induced increase in HbR. We find that the HbT-dip counters spiking induced HbR increases, imposing an upper-limit to HbR concentration in the capillaries. Building on our results, we explore the possibility of active venule dilation (purging) as a possible mechanism for the HbT dip.

## 1. Introduction

Functional neuroimaging is a powerful non-invasive tool for studying brain function in health and disease that uses changes in blood oxygenation as a proxy for estimating local neuronal activity (Logothetis, 2008). However, which feature of the hemodynamic signal best reflects local neuronal activity still remains an open question. The most commonly used feature is the hemodynamic response amplitude, which is slow and unspecific, given that it can reflect both excitatory and inhibitory neuronal activity (Logothetis et al., 2001; Logothetis, 2008). Since neuronal processes such as multi-unit spiking are fast, dynamic, and spatially localized, a feature in the BOLD signal with similar properties, which also correlates strongly with local spiking activity, would be an ideal candidate. Early fMRI studies reported such a quick and localized dip in the initial BOLD signal immediately following stimulus onset in various brain areas (Yacoub and Hu, 2001; Hu and Yacoub, 2012). This early decrease was termed the “initial-dip”, and was believed to originate from a rise in deoxy-hemoglobin (HbR) caused by stimulus-induced changes in localized neuronal activity (Hu and Yacoub, 2012). Supporting evidence comes from studies reporting spatially localized dips in tissue partial oxygen pressure (Parpaleix et al., 2013; Zhang et al., 2015), and increases in the concentration of HbR, observed at the time of the dip. The initial-dip is also more spatially localized than the positive BOLD response (Watanabe et al., 2013), and has been used to accurately map orientation columns in the visual cortex better than the positive-response (Kim et al., 2000). Based on these observations, the initial-dip is believed to represent focal neuronal activity (Kim et al., 2000). Although the initial-dip has been observed in various functional neuroimaging modalities, such as BOLD-fMRI (Hu and Yacoub, 2012; Watanabe et al., 2013; Siero et al., 2015), optical imaging (Sirotin et al., 2009; Tian et al., 2010), fNIRS (Zaidi et al., 2015), and partial oxygen pressure (p_O2_) measurements (Parpaleix et al., 2013), its vascular origins are uncertain and its precise neuronal correlates are disputed.

**Figure 1:**
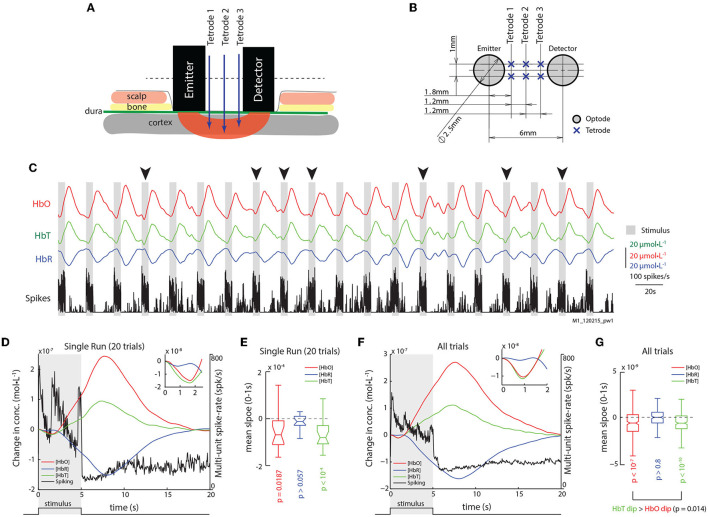
Epidurally measured fNIRS measurements reveal initial dips in hemodynamic signals. **(A)** Illustration of the sensor array with placement of fNIRS optodes and electrodes relative to scalp and brain tissue. **(B)** Transverse section of the sensor array with distances between optodes and electrodes. See Section 2 for details. **(C)** Traces of HbO, HbR, and Spiking from an example run with 20 trials. Gray bars represent epochs of visual stimulation. Arrows mark trials where initial dips are obvious in signal trends. **(D)** The mean traces of HbO, HbR, HbT, and multi-unit spiking (units on the right) for trials shown in **(C)**. (Inset) Same hemodynamic traces, but from 0 to 2.5 s. The initial dip is observed in the HbO and HbT (inset), but not in the HbR traces. The shaded region represents visual stimulus presentation. **(E)** Distribution of slopes from 0 to 1 s for HbO, HbR, and HbT traces for trials in **(C)**. The distributions of HbO and HbT slopes are less than zero, but not for those for HbR (*p*_*HbO*_ = 0.0187; *p*_*HbT*_ < 10^−4^; *p*^*HbR*^ = 0.099; *n* = 20; Š). **(F)** The mean traces of HbO, HbR, HbT, and multi-unit spiking activity (units on the right) for all trials. (Inset) Same hemodynamic traces, but from 0 to 2 s. **(G)** Distribution of signal slopes from 0 to 1s for HbO, HbR, and HbT traces for all trials. The distributions for HbO and HbT are less than zero, but not for HbR (*p*_*HbO*_ < 10^−7^; *p*_*HbT*_ < 10^−10^; *p*_*HbR*_ > 0.1; *n* = 260). However, HbT dips were stronger than HbO dips (*p* = 0.014).

We recently documented a method for the simultaneous acquisition of epidural fNIRS and intra-cortical electrophysiology in primates (Figures 1A, B), demonstrating that fNIRS has high SNR when acquired epidurally (Zaidi et al., 2015), making it ideal for studying local neurovascular interactions. FNIRS uses a light-emitter and detector pair (optode pair) to measure changes in concentrations of oxygenated (HbO), deoxygenated (HbR) and total (HbT) hemoglobin, within the vascular compartments in a small volume of tissue (Villringer and Chance, 1997; Ferrari and Quaresima, 2012). Using this setup, we recorded both spontaneous and stimulus-induced activity in the primary visual cortex of two anesthetized monkeys.

## 2. Methods

### 2.1. Data collection

#### 2.1.1. Surgery and craniotomy

Two healthy adult monkeys, M1 (female; 8 kg) and M2 (male; 10 kg), were used for the experiments. All vital parameters were monitored during anesthesia. After sedation of the animals using ketamine (15 mg/kg), anesthesia was initiated with fentanyl (31 μg/kg), thiopental (5 mg/kg), and succinylcholine chloride (3 mg/kg), and then the animals were intubated and ventilated. A Servo Ventilator 900C (Siemens, Germany) was used for ventilation, with respiration parameters adjusted to each animal's age and weight. Anesthesia was maintained using remifentanil (0.2-1 μg/kg/min) and mivacurium chloride (4–7 mg/kg/h). An iso-osmotic solution (Jonosteril, Fresenius Kabi, Germany) was infused at a rate of 10 ml/kg/h. During the entire experiment, each animal's body temperature was maintained between 38.5 and 39.5°C, and SpO_2_ was maintained above 95%. Under anesthesia, a craniotomy was made on the left hemisphere of the skull to access the primary visual cortex. During each experiment, the bone was removed to create a rectangular slit measuring 3 mm anterio-posteriorly and 20 mm medio-laterally, exposing the dura. Connective tissue, if present above the dura, was carefully removed. For each monkey, at least 2 weeks were allowed to pass between successive experiments. All protocols were approved by the local authorities (Regierungspräsidium, Tübingen) and are in agreement with European guidelines for the ethical treatment of laboratory animals.

#### 2.1.2. Near-infrared spectroscopy

We used a NIRScout machine purchased from NIRx Medizintechnik GmbH, Berlin. The system performs dual wavelength LED light-based spectroscopic measurements. The wavelengths used were 760 and 850 nm, with a maximum of 5 μW effective power at the emitter end. Sampling was performed at 20 Hz. We used modified emitters and detectors, and optical fiber bundles for sending the light from the LED source into the tissue, and also for detecting refracted light from the tissue. The fiber bundles were ordered from NIRx Medizintechnik GmbH, Berlin, Germany. Both the emitter and detector fiber bundles had iron ferrule tips with an aperture of 2.5 mm on the ends that touched the dura. We used three optodes in a linear arrangement separated by 6 mm each. Three tetrodes and single-wire electrodes each were added between each pair of adjacent optodes. We used the central optode as a constant detector, and alternated the peripheral optodes during sessions, such that on a given experimental day, 50% of data came from one emitter-detector pair and 50% from the other. The recording instrument was connected via USB to a laptop computer running an interactive software called NIRStar provided along with the instrument. The software was used for starting and stopping recordings, and also for setting up the various parameters, such as the number of sources and detectors, and the sampling rate. The instrument received TTL pulses from the stimulus system and the electrophysiological recording system, for synchronization purposes. The system sent 1 ms TTL pulses every 50 ms to the recording system that corresponded to light pulses.

#### 2.1.3. Electrophysiology

Custom built tetrodes and electrodes were used. All tetrodes and single electrodes had impedance values less than 1 MΩ. The impedance of each channel was noted before loading the tetrodes on to the drive, and once again while unloading the tetrode after the experiment, to ensure that the contacts were intact throughout the duration of the experiment. To drive the electrodes into the brain a 64-channel Eckhorn matrix was used (Thomas Recording GmbH, Giessen, Germany). The electrodes were loaded in guide tubes a day before the experiment. On the day of the experiment, the tetrodes were driven using a software interface provided by Thomas Recording GmbH, Giessen, Germany. The output was connected to a speaker and an oscilloscope, with a switch to help cycle between different channels. We advanced electrodes into the cortex one by one until we heard a reliable population response to a rotating checkerboard flickering at 0.5 Hz.

#### 2.1.4. Spontaneous activity

For each run, spontaneous activity was recorded for 15 min, in the absence of visual stimulation. The eyes of the monkey were closed and thick cotton gauze was used to cover the eyelids. Visual stimulation A fundus camera was used to locate the fovea for each eye. For presenting visual stimulation, a fiber optic system (Avotec, Silent Vision, USA) was positioned in front of each eye, so as to be centered on the fovea. To adjust the plane of focus, contact lenses (hard PMMA lenses, Wöhlk, Kiel, Germany) were inserted to the monkey's eyes. We used whole-field, rotating chequerboard to drive the neural activity. The direction of rotation was reversed every second. Each trial consisted of 5 s of visual stimulation followed by 15 s of a dark screen. A single run consisted of 20 trials. Data presented are from 13 runs spread over 8 experimental days. Signal processing and data analysis All analyses were performed in MATLAB using custom written code. Only runs that cleared visual screening for artifacts were used. Data points that were larger than 5 SDU were excluded from the analysis, so as to avoid tail-effects for correlation analysis. Normality for each distribution was confirmed before analysis was performed.

### 2.2. Data analysis

#### 2.2.1. fNIRS signal processing

The raw wavelength absorption data from the NIRS system was converted to concentration changes of [HbO] and [HbR] using custom matlab script based on the modified Beer-Lambert equation (DPF = 6,6), with molar extinction coefficients derived from Wray et al. (1988). For correlating hemodynamic signals with neural activity, the signals were band-pass filtered between 0.01 and 0.1 Hz to remove any low frequency signal drifts and cardiac-pulsations. For a trial-by-trial analysis, the hemodynamic response for each trial was zero-corrected by subtracting, from each hemodynamic response, the value at the start of the trial.

#### 2.2.2. Electrophysiological signal processing

The extracellular field potential signal was recorded at 20.8333 kHz and digitized using a 16-bit AD converted. From the raw signal, eight frequency bands [namely, DeltaTheta (1–8 Hz), Alpha (9–15 Hz), Spindle (15–20 Hz), low Gamma (20–40 Hz), Gamma (40–60 Hz), high Gamma (60–120 Hz), very high Gamma (120–250 Hz), and MUA (1–3 kHz)] were band-pass filtered using a 10th order Butterworth filter. The choice of bands was made to enable a comparison with previous studies, specifically (Goense and Logothetis, 2008). The envelope for each band was then obtained by taking the absolute value of the Hilbert transform of the filtered signal. The band-envelope was then converted to standard deviation units by subtracting the mean and dividing by the standard deviation of the signal. This signal was then resampled at 20 Hz, to allow comparisons with hemodynamic signals. 290 Spike rates were obtained by detecting peaks in the MUA signal larger than a threshold (2 SDU), and by counting the threshold-crossing events in 50 ms bins. Varying the detection threshold between 2, 3, or 4 SDU did not affect our overall results.

#### 2.2.3. System identification based impulse response estimation in spontaneous activity

Impulse response functions from spiking to HbO, HbR, and HbT were obtained using the system identification toolbox in Matlab. Each hemodynamic signal was first filtered between 0.01 and 1 Hz and then normalized by subtracting the mean 294 and dividing by the standard deviation. Spike-counts were obtained by counting the number of threshold crosses (≥3 SDU) in the 1–3 kHz band in 50 ms bins. The bin-count was then divided by the length of the time window to obtain spike rates in spikes/s. Each spike-rate trace was then smoothened by convolving with a Gaussian function of unit amplitude and 100 ms standard deviation. The 900 s (15 min) recordings were divided into four epochs of 225 s each. Each timeseries (for each band and the hemodynamic traces) was then resampled at 1 Hz. The reason for this was the need to implement a whitening filter to remove auto-correlations from the electrophysiology signal. In order enable comparison across such a wide range of frequencies (between 1 and 1,000 Hz), the signal was resampled at 1 Hz. For estimating the impulse response function for each epoch, the electrophysiology timeseries for that epoch was used as the input and the hemodynamic signal as output.

#### 2.2.4. Calculation of modulation indices

The “On” epoch for each trial was defined as the time from 0 to 5.05 s. The extra 0.05 s were added to accommodate for the off response. The “Off” epoch was defined as the time between 5.05 and 10.05 s. The modulation index (MI) was then calculated using the formula:


$$\text{neural modulation index (MI)}=\frac{\left(SR_{On}-SR_{\text{Off}}\right)}{\left(SR_{\text{On}}+SR_{\text{Off}}\right)}$$

where **SR**_On_ is the mean spike-rate during Stim On, and **SR**_Off_ is the mean spike-rate during Stim Off for each trial. Runs without significant visual modulation of spike-rates were excluded from the analysis.

#### 2.2.5. Statistics

All distributions were confirmed to be normally distributed using the Kolmogorov-Smirnov test in Matlab, before using means as a measure of central tendency. All correlation coefficients represent Pearson's correlation coefficient and corresponding significance values. To calculate the correlations, the trials were sorted and divided into 10 bins (with 26 trials per bin). The mean values of each bin were then correlated. This was done to avoid an otherwise large trial-by-trial variation. The results were independent of the number of bins used for correlation analysis (see Supplementary Figure 1)

## 3. Results

Figure 1C shows the traces of HbO, HbR, HbT, and multi-unit spike-rates for an example run with visual stimulation, consisting of 20 trials. The gray bars mark the 5 s of visual stimulation (a whole-field rotating checkerboard with high contrast), followed by 15 s of rest (white spaces). Obvious dips in the HbO signals can be observed on some trials (black arrows). The average traces of these 20 trials elicited observable dips in both HbO and HbT (Figure 1D). We used the mean signal slope within the first second after stimulus onset as a metric of the “strength” of the initial-dip for each hemodynamic signal, and found that both the HbO and HbT traces had significant dips (Figure 1E). Similarly, in the mean traces of all 260 trials from our dataset, a clear dip in the HbO and HbT signals can be observed, without any changes in HbR (Figures 1F, G and Supplementary Table 1).

**Figure 2:**
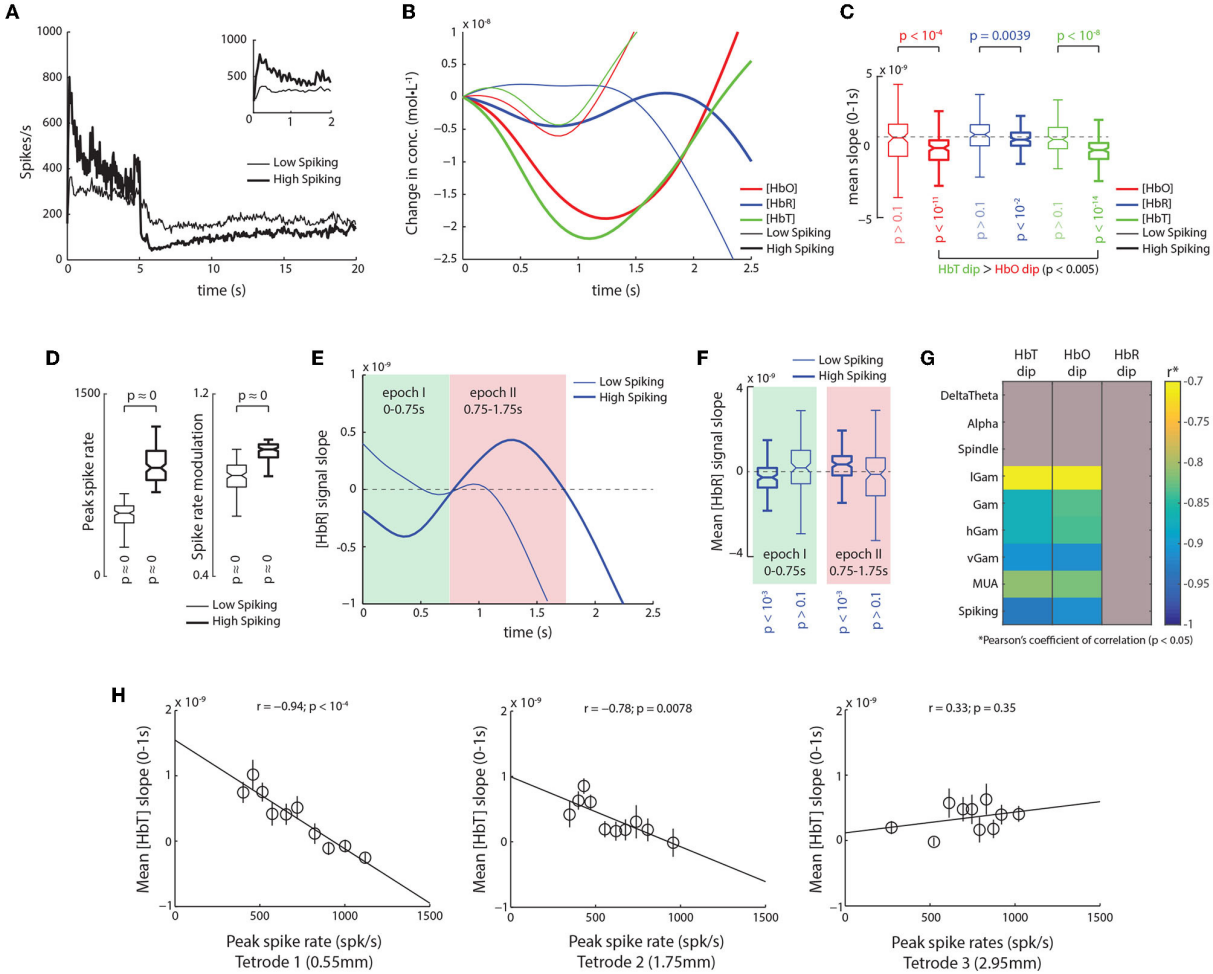
Trials with high spiking activity reveal initial dips comprise of an early HbT decrease, and late HbR increase. **(A)** Mean traces of spike-rates for trials with high and low spiking immediately after stimulus onset (thick and thin traces, respectively). (Inset) Same traces, but between 0 and 2 s. **(B)** Mean traces of hemodynamic signals for trials with low (thin) and high spiking as shown in **(A)**. A clear increase in the dips is observed for high spiking trials with the largest dips elicited in HbT traces. **(C)** Average slopes from 0 to 1 s for HbO, HbR, and HbT traces for high (thick) and low (thin) spiking trials. HbO, HbR, and HbT all elicit significant dips in high-spiking trials (*p*_*HbO*_ < 10^−11^; *p*_*HbT*_ < 10^−14^; *p*^*HbR*^ < 10^−2^; *n* = 128; Š), with larger dips in HbT than HbO (*p* < 0.005; *n* = 125; pairwise Ś). Interestingly, trials with low spiking trials do not elicit significant dips in either HbO, HbR, or HbT (*p* < 0.1; *n* = 122; Š). **(D)** Distribution of peak spike-rates and visual modulation of spike-rates for trials with high (thick) and low (thin) peak spike-rates. This illustrates that even though the peak rates were lower in the low-spiking trials, the overall spiking activity was significantly high, as was the visual stimulus induced modulation of spike rates (see Section 2 for calculation of modulation index). **(E)** Analysis of the slope of HbR traces in high spiking trials reveals a biphasic response, which is almost all but absent in low spiking trials. In high spiking trials (thick trace), an initial negative slope is observed roughly between 0 and 0.75 s (epoch I, shaded green), followed a positive slope roughly between 0.75 and 1.75 s (epoch II, shaded red). **(F)** For high spiking trials, the distribution of mean HbR slopes were significantly negative during epoch I (*p* < 10^−3^; *n* = 125; Ś), and significantly positive during epoch II (*p* < 10^−3^; *n* = 125; Š). In contrast, low spiking trials showed no significant modulation of HbR slopes during either epoch I or II (*p* > 0.1; *n* = 122; Š). **(G)** Correlation of mean dips in HbT, HbO, and HbR stimulus induced peaks in the power of various LFP frequencies bands and Spiking. Correlations with *p* > 0.05 are grayed. Only high frequency bands showed a significant correlation with initial dips, with spiking activity eliciting the strongest relationship, that were marginally higher for HbT than HbO. **(H)** Strength of the relationship between the HbT dip and spiking activity decreases with distance from the NIRS emitter. Strongest correlations are observed on tetrode closest to emitter (0.55 mm away from emitter edge, 1.8 mm from emitter center), whereas no correlations are observed on tetrode 2.95 mm away (4.2 mm from center).

To understand the relationship between neuronal activity and the initial-dip, we divided the 260 trials in our dataset into two groups based on the peak spike-rate during stimulus onset, namely, high-spiking trials (899.96 ± 12.89 spk/s; *n* = 122) and low spiking trials (497.28 ± 8.35 spk/s; *n* = 125) (Figure 2A). For high-spiking trials we observed strong dips in HbO, HbR, and HbT traces. Although the overall distributions of dips in HbO and HbT were not significantly different from one another (*p* > 0.32; Wilcoxon's rank-sum test), a trial-by-trial comparison revealed that HbT dips were in fact larger than corresponding HbO dips (*p* < 10^-5^, Wilcoxon's signed-rank test; Figures 2B, C). Although low spiking trials also seem to elicit faint modulations in the HbO and HbT signals, we did not observe significant decreases in their slopes (*p* < 0.1; Figure 2C). The low-spiking trials did, however, have both significantly high peak spike-rates and strong stimulus-induced spike-rate modulations (Figure 2D), meaning even though significant bursts in spiking occurred during these trials, they weren't strong enough to elicit dips in the HbO, HbR, or HbT signals. Furthermore, in high-spiking trials, we observed a biphasic response in the slope of HbR signal, which was again absent in the low-spiking trials (Figure 2E). In high spiking trials, the HbR signal elicited a small albeit significant dip between 0 and 0.75 s post stimulus onset (which we called epoch I) and a later rebound between 0.75 and 1.75 s (called epoch II, Figure 2F). This illustrates that there is indeed an increase in HbR signal with higher spiking activity, which is absent during low spiking. Overall, these results suggest that early onset modulations (HbO and HbT initial-dips and HbR-rises) observed in the various hemodynamic signals are detectable only during high spiking activity, and that low spiking activity, even though significantly modulated in itself, failed to elicit detectable changes in the corresponding hemodynamic signals.

We next assessed the relationship of the initial-dip with various bands of the local field potential (LFP). We filtered the broadband signal into eight frequency bands, namely the DeltaTheta (1–8 Hz), Alpha (9–15 Hz), Spindle (15–20 Hz), low-Gamma (lGam, 20–40 Hz), Gamma (Gam, 40–60 Hz), high-Gamma (hGam, 60–100 Hz), very high-Gamma (vGam, 125–300 Hz), and multi-unit activity (MUA, 1–3 kHz), and obtained their respective band envelopes (see Section 2 for details on choice of bands). From these LFP bands, only peaks in high-frequency bands had significant correlations with the HbO and HbT dips (Figure 2G). The strongest dependencies, however, were still observed with spiking activity for both HbO and HbT dips, with slightly higher correlations observed with HbT than for HbO (Figure 2G and Supplementary Figure 1). These observations reveal that the initial-dip is elicited only by excitatory neuronal activity such as spiking and high frequency LFPs (specifically in the gamma bands). We next determined how this relationship with spiking varied as a function of distance over cortical surface. We obtained the correlations between the HbT dip and the peak spike-rates on the three tetrodes placed between the emitter and detector (Figures 1A, B). We found that the correlation was strongest with the tetrode closest to the emitter (*r* = −0.94; *p* < 10^-4^, Figure 2H), and that this relationship decreased with increasing distance from the emitter. The results were identical when we used the peak-amplitude of the initial-dip instead of the mean signal slope (Supplementary Figure 2). Interestingly, the overall spike-rate modulation was almost the same across all three tetrodes (Tetrode1 = 0.887 ± 0.006, Tetrode2 = 0.838 ± 0.006, Tetrode3 = 0.832 ± 0.012), meaning that the difference in correlations were not based on differences in spike-rates. This finding not only corroborates the idea that the initial-dip is a highly localized hemodynamic response, but also suggests that fNIRS might have a spatial sampling bias in favor of the emitter, questioning the popular “banana model” that assumes uniform sampling through the volume of tissue between the emitter and detector (Villringer and Chance, 1997). Together, these sets of results demonstrate that the HbO and HbT dips reflect strong, focal excitatory neuronal activity.

**Figure 3:**
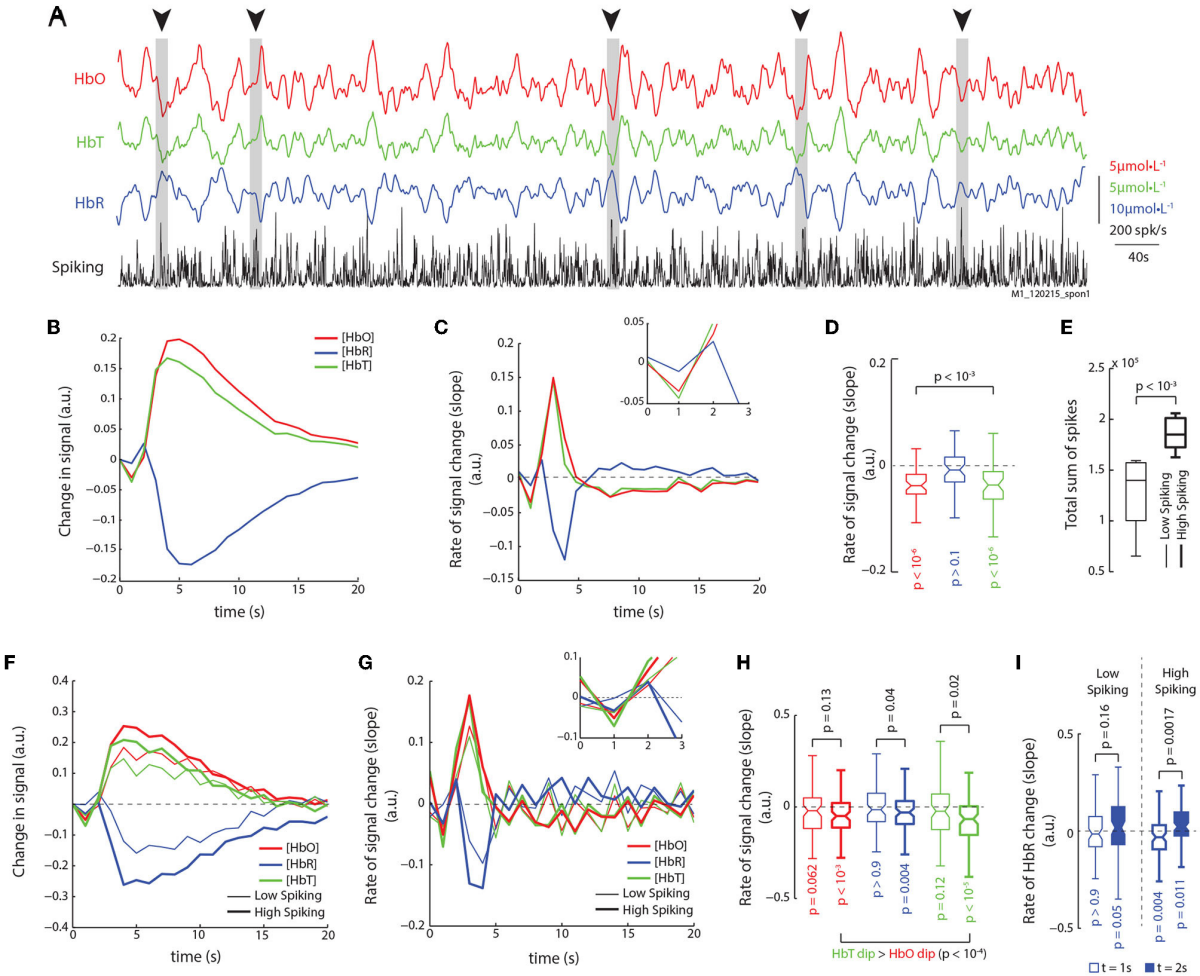
Analysis of spontaneous activity in the absence of visual stimulation reveals identical relationships. **(A)** Traces of HbO, HbR, HbT, and spike-rates from an example run of 900 s. Periods of high spiking activity that elicit an observable dip in HbO and HbT are marked with arrows and gray bars. **(B)** We used system identification to estimate the impulse response functions from spiking to HbO, HbR, and HbT signals in recordings of spontaneous activity. The mean impulse response reveals a dip in HbO and HbT (mean of 48 impulse response functions obtained from 16 runs lasting 900 s each; see Section 2 for details). **(C)** Rate of change of the impulse response functions for HbO, HbR, and HbT reveals dips in both HbO and HbT, and a late rise in the HbR. (Inset) Same traces but between 0 and 3 s. **(D)** Distribution of slopes for HbO, HbR, and HbT at *t* = 1 s. Only HbO and HbT have significant dips, but not HbR (Š; *n* = 48). **(E)** The runs were divided based on the total sum of spikes in each run, and separated into low spiking and high spiking runs. High spiking runs had significantly higher spike sums (Ś; *n* = 8). **(F)** The mean impulse responses for high and low spiking runs reveal stronger modulation of hemodynamic signals on high spiking trials. Color-code same in following figures. **(G)** Mean traces of slopes of impulse responses shown in **(F)**. High spiking trials elicit an obvious dip at *t* = 1 s. (Inset) Same traces but from 0 to 3 s. **(H)** Distribution of dips for low and high spiking trials (legend same as **F**). Only high spiking trials have significant dips in all three signals. Also, HbT dips were larger than HbO dips (Ś; *n* = 80). **(I)** When comparing the HbR dip and rebound at *t* = 1 s and *t* = 2 s, resp., only high spiking trials reveal a strong dip and rebound in the HbR signal.

It might be argued that stimulus induced activity introduces artificial correlations between the neuronal and hemodynamic responses, by inducing highly synchronous patterns of activations in both sets of signals Logothetis et al. (2001). To ensure our results didn't arise from such correlations, we analyzed recordings of spontaneous ongoing activity in the absence of visual stimulation, where the monkeys' eyes were closed and covered with thick gauze. Figure 3A shows traces of HbO, HbR, HbT, and spike-rates for an example run of spontaneous activity, consisting of 15 min. Dips in the HbO and HbT signals (sudden decreases in the traces of concentration) can be seen to coincide with strong bursts in spiking activity (Figure 3A, arrows and bars). To analyze the relationship between spiking and hemodynamic signals, we used system identification techniques to estimate the impulse response from spiking to HbO, HbR, and HbT traces. This method uses the Wiener-Hopf relationship (Keesman, 2011) to estimate the impulse-response (IR) to a unit-pulse (unit amplitude and duration at *t* = 0) from the input (spiking activity) on the output (hemodynamic signal), and is independent of the shape and auto-correlation structure of the input. Figure 3B shows the mean impulse-responses from spiking on HbO, HbR, and HbT traces. There is an evident dip in the HbO and HbT traces. The rate of change of the impulse responses (ΔIR, difference of consecutive values) provides a clearer picture of the signal dynamics (Figure 3C), where a decrease in HbO and HbT can be observed at *t* = 1 s (Figure 3D; *n* = 48; Wilcoxon's one-tailed signed-rank test), and a late rebound of HbR can be observed at *t* = 2 s. We next used the total spike count in each run to separate the runs into high-spiking and low-spiking runs (Figure 3E). Figure 3F shows the impulse-responses obtained for the high-spiking (thick traces) and low-spiking (thin traces) runs. The ΔIRs show that the high-spiking trials had large, significant dips for all three hemodynamic signals (HbO, HbR, and HbT; Figures 3G, H). Furthermore, we found no significant difference between the overall distributions of HbO and HbT dips (*p* > 0.45, *n* = 85, Wilcoxon's rank-sum test). However, on a trial-by-trial comparison, again HbT dips were significantly stronger than HbO-dips (Figure 3H; *p* < 10^-4^, *n* = 85, Wilcoxon's one-tailed signed-rank test). Interestingly, even though the low spiking runs seemed to elicit dips as well, they did not reach significance in our data. Furthermore, only the high-spiking runs elicited significant modulations in HbR, with both significant dips at *t* = 1 s, and rebounds at *t* = 2 s (Figure 3I). These results are identical to those obtained from the analysis of stimulus induced activity, and are thus independent of the visual stimulation paradigm.

In the analysis of both spontaneous and stimulus-induced activity, we find that the initial-dip is dominated by a decrease in HbT, in trials with strong bursts in spike-rates. However, it might be argued that this decrease in HbT is not an actual change in blood volume, but a consequence arising from signal trends, such as the slope of the hemodynamic signal before stimulus onset, or from the choice of analysis parameters, such as the differential path-length factors (DPFs) used for the conversion of optical densities to changes in concentration (the only parameter-dependent transformation in the analysis of fNIRS; Villringer and Chance, 1997). Surprisingly, the strength of the initial-dip failed to correlate with the mean slope of the hemodynamic signal 0–2 s prior to stimulus onset (*r* = −0.002, *p* > 0.9, Pearson's coefficient of correlation), suggesting that the trend of the hemodynamic signal before the dip fails to affect the size of initial-dip in any significant way. We also used various combinations of physiologically relevant DPFs (as reported in earlier studies, Jasdzewski et al., 2003) in the estimation of concentration changes in HbO, HbR, and HbT, and obtained identical results (see Supplementary Figure 3). We also analyzed the raw signals of optical density changes for both wavelengths (760 and 850 nm). We found a significant decrease in the optical densities for both chromophores, which was enhanced in high-spiking trials (see Supplementary Figure 4), demonstrating that the effects are present in the raw recordings of the hemodynamic changes and do not arise as a consequence of the conversion from changes in absorption to changes in concentration. Furthermore, within trials with low spiking activity, even though we do not see significant changes in the slope of the HbO and HbR signals, we do find small but significant changes in their concentration (see Supplementary Figure 5). We detected significant increases in the HbR concentration (mean HbR concentration change between 0 and 0.8 s, *p* < 0.05, Wilcoxon's signed-rank test) as well as significant decreases in the HbO concentration (see Supplementary Figure 5C, *p* < 0.05; *n* = 125; Wilcoxon's sign-rank test) but failed to detect significant changes in HbT (*p* > 0.1, *n* = 125; Wilcoxon's signed rank test), an observation that is in agreement with previous reports on the initial-dip (Jasdzewski et al., 2003; Martin et al., 2006). Finally, the trials within the lowest quartile of spike-rates elicited neither initial-dips (in HbO, HbR, or HbT traces, mean slope between 0 and 1 s), nor changes in concentration (mean concentration change between 0 and 1 s), even though these trials still had significantly high bursts in spike rates (peak rate 300 ± 40 spk/s; *p* < 10^-11^; *n* = 59, Wilcoxon's signed-rank test). These observations demonstrate two different manifestations of the initial-dip. During low-spiking, there is an oxymetric change consisting of increases in HbR and decreases in HbO concentration. During very high spiking, there is a large volumetric change, consisting of a decrease in HbT (and consequently HbO and HbR).

Interestingly, all significant dips detected in optical density traces also translated to significant changes in hemoglobin concentration, irrespective of the choice of parameters used for converting optical density to concentration change (see Supplementary Figure 3), specifically the differential path length factor (dpf). Combined with previous studies (Jasdzewski et al., 2003), these results disagree with an earlier report where changes in optical density failed to translate to changes in HbO, HbR, or HbT signals (Sirotin et al., 2009).

## 4. Discussion

While the exact vascular compartments that fNIRS samples from have not yet been firmly established, it is generally believed to reflect oxymetric changes within vessels smaller than 1 mm in diameter (Ferrari and Quaresima, 2012), such as pre-capillary arterioles, capillaries, and post-capillary venules (Capillary and Peri-capillary Vessels, henceforth CPVs), which is where most of the oxygen-exchange with neuronal tissue occurs (Sakadžić et al., 2014). In our data, the HbO/HbT dip ratio (the ratio of HbO to HbT decrease at maximal dip) is 50.4 ± 17% (mean ± sem) for stimulus-induced, and 58.9 ± 32% for spontaneous activity, which is within the range of oxygen saturation reported within CPVs (Sakadžić et al., 2014). Furthermore, in our recordings the largest changes occur in the HbO signal and not HbT (Figures 2D, F) during the hemodynamic response, even though the overall blood volume in cortical tissue increases post arteriole dilation, further demonstrating that our recordings mostly reflect concentration changes in the CPVs.

A possible means to attain an immediate decrease in CPV blood volume (HbT) could be through the active dilation of post-capillary venules, triggered by very strong bursts in spiking activity. While such a phenomenon has not been documented as yet, post-capillary venules have been shown to have band-like smooth muscles encircling their circumference, similar to those associated with pre-capillary arterioles (Hill et al., 2015). Furthermore, although not explicitly mentioned or discussed in their report, small venules can be seen to increase their diameter almost simultaneously with strong arteriolar dilation in recordings of spontaneous activity (see Figures 2A, B in Drew et al., 2011). Since the primary arterioles that dilate in response to neuronal activity are further away from the capillaries, the influx of blood takes longer to reach the capillaries, and hence “ballooning” (Devor et al., 2011; Buxton, 2012) does not explain the near instantaneous dilation of the post-capillary venules. However, in the case of active post-capillary venule dilation, the decrease in capillary blood pressure would briefly compress the capillary, flushing the blood out, before the influx of oxygenated blood caused by arteriole dilation, effectively purging the HbR-rich capillary blood. We refer to this phenomenon as purging. Recently, erythrocytes have been reported to deform with reduced oxygen tension (*p*O_2_), facilitating an increase in their flow-rate through the capillary lumen (Wei et al., 2016), aiding the quick removal of HbR from capillaries. Indeed, a transient increase in capillary RBC velocity can also be observed immediately after stimulus onset, which briefly subsides, before finally increasing again (see Figures 4B, D in Drew et al., 2011), consistent with our suggested mechanism where a very early HbT decrease occurs due to venule dilation, flushing out the HbT, followed by a delayed HbT increase caused by arteriole dilation.

**Figure 4:**
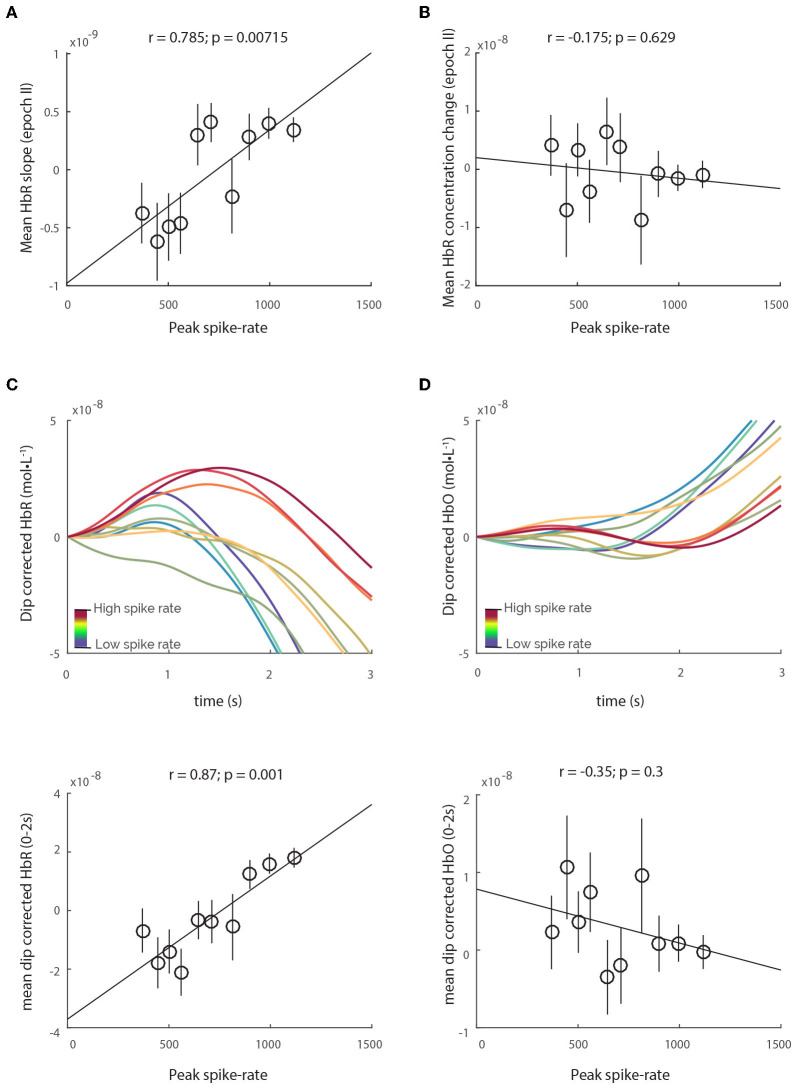
HbR-rebound does not lead to increase in HbR concentration. Although there is a correlation between spiking and the mean HbR slope in epoch II **(A)**, the relative HbR concentration change remains unchanged with spiking **(B)**. **(C)** Dip-corrected HbR traces, obtained by simply subtracting the HbT traces from HbR reveals obvious increases in HbR concentration that correlate with spiking. However, no such relationship is observed with dip-corrected HbO traces **(D)**.

A natural question that arises is whether purging actually serves any physiological purpose. One possible purpose could be to prevent HbR accumulation in the capillaries, enforcing an “upper-limit” of HbR concentration by flushing the blood inside capillary lumen, as well as facilitating the influx of oxygen saturated blood in the proximal arterioles, before distal arteriole dilation can resupply the capillaries. Accordingly, in our analysis we found that spiking correlated strongly with the HbR-rebound (Figure 4A), which is the increase in HbR post neuronal activity. However, the HbT dips were still consistently larger than the HbR-rebound, maintaining an effective upper-limit of HbR concentration, and hence no relationship was observed between spiking and HbR concentration change between 0.75 and 1.75 s (Figure 4B). Moreover, when corrected for the HbT dip (by subtracting HbT traces from HbR traces), the “dip-corrected” HbR traces reveal increases in concentration that are strongly correlated to spiking activity (Figure 4C). In contrast, no such relationship is observed with “dip-corrected” HbO traces and spiking activity (Figure 4D), illustrating that the initial-dip efficiently counters rising HbR concentration in the vascular tissue. This deoxygenated blood flushed from individual CPVs would flow into the surface venules, transiently increasing their HbR concentration. An illustration of the proposed mechanism for purging, along with its effects on both vascular and hemo-dynamics can be seen in Supplementary Figure 6. Indeed, cortical-depth resolved BOLD-fMRI, believed to reflect changes in tissue HbR (Logothetis, 2008; Huettel et al., 2014), reveals that the amplitude of the initial-dip is higher near the cortical surface, in both human (Siero et al., 2015) and animal (Tian et al., 2010) studies, where the least amount of tissue-oxygen exchange occurs, but where the HbR saturated HbT accumulates post venule dilation. Further experiments quantifying changes in HbO and HbR in the various vascular compartments could shed more light on the exact vascular mechanisms of the initial-dip. Nevertheless, our results conclusively demonstrate that the initial-dips in both HbO and HbT traces are strongly correlated with highly localized spiking activity. Furthermore, since we find no relationships between the initial-dip and low-frequency LFP activity, this demonstrates that the initial-dip is a highly specific marker of localized bursts in spiking activity.

We also find that fNIRS represents focal neurovascular changes close to the emitter, challenging the generally accepted “banana” model that assumes the cortical volume sampled by fNIRS to be uniform between the emitter and detector (Ferrari and Quaresima, 2012). Concurrently, a study comparing simultaneously recorded fNIRS and fMRI signals in humans finds that the voxels correlating best with HbO/HbR changes are consistently closer to the emitter (see Figure 2 and Table 2 in Cui et al., 2011), though this is not explicitly stated in the results or discussion. Overall these results shed further light on the neurovascular changes underlying the initial-dip, and enable a much better interpretation of functional neuroimaging signals by providing evidence for a unique, fast marker of spiking activity.

Finally, these results are based on recordings from anesthetized monkeys, and while it can be argued that these results may not apply to the awake preparation, it has been demonstrated that this anesthesia regime does not significantly alter local neurovascular coupling in the primary visual cortex (Goense and Logothetis, 2008), which is the site of our recordings. We also followed earlier preparation and anesthesia protocols as closely as possible, to ensure our results could be interpreted and contrasted with previous work (Logothetis et al., 2001; Goense and Logothetis, 2008).

In conclusion, we show that the initial dip consists of both oxymetric (HbO and HbR) and volumetric changes (HbT) in vascular tissue, but is dominated by HbT changes (volumetric) that are correlated to highly-localized spiking activity, demonstrating that these changes are specific to focal, excitatory neuronal activity. We further show that the HbT dips exactly counteract the transient increases in HbR caused by the neuronal activity. Based on the temporal dynamics of the HbT dip, we propose that these instantaneous decreases of HbT could be caused by active venule dilation (purging) triggered by strong spiking activity. These results reconcile conflicting reports on the vascular mechanisms and neuronal correlates of the initial dip (Buxton, 2001; Sirotin et al., 2009; Uludağ, 2010; Siero et al., 2015).

## Data availability statement

The original contributions presented in the study are included in the article/Supplementary material, further inquiries can be directed to the corresponding author.

## Ethics statement

All protocols were approved by the local authorities (Regierungspräsidium, Tübingen) and are in agreement with European guidelines for the ethical treatment of laboratory animals.

## Author contributions

AZ, NB, NL, EF, and RS contributed to conception and design of the study. AZ collected and analyzed the data and wrote the first draft of the manuscript. All authors contributed to manuscript revision, read, and approved the submitted version.
