# Supplementary material for: The hemodynamic initial-dip consists of both volumetric and oxymetric changes reflecting localized spiking activity
Source: Front Neurosci. 2023 May 25;17:1170401. doi: 10.3389/fnins.2023.1170401 (PMC10248142; doi:10.3389/fnins.2023.1170401)
Supplement: Supplementary file 1 [file Data_Sheet_1.pdf]

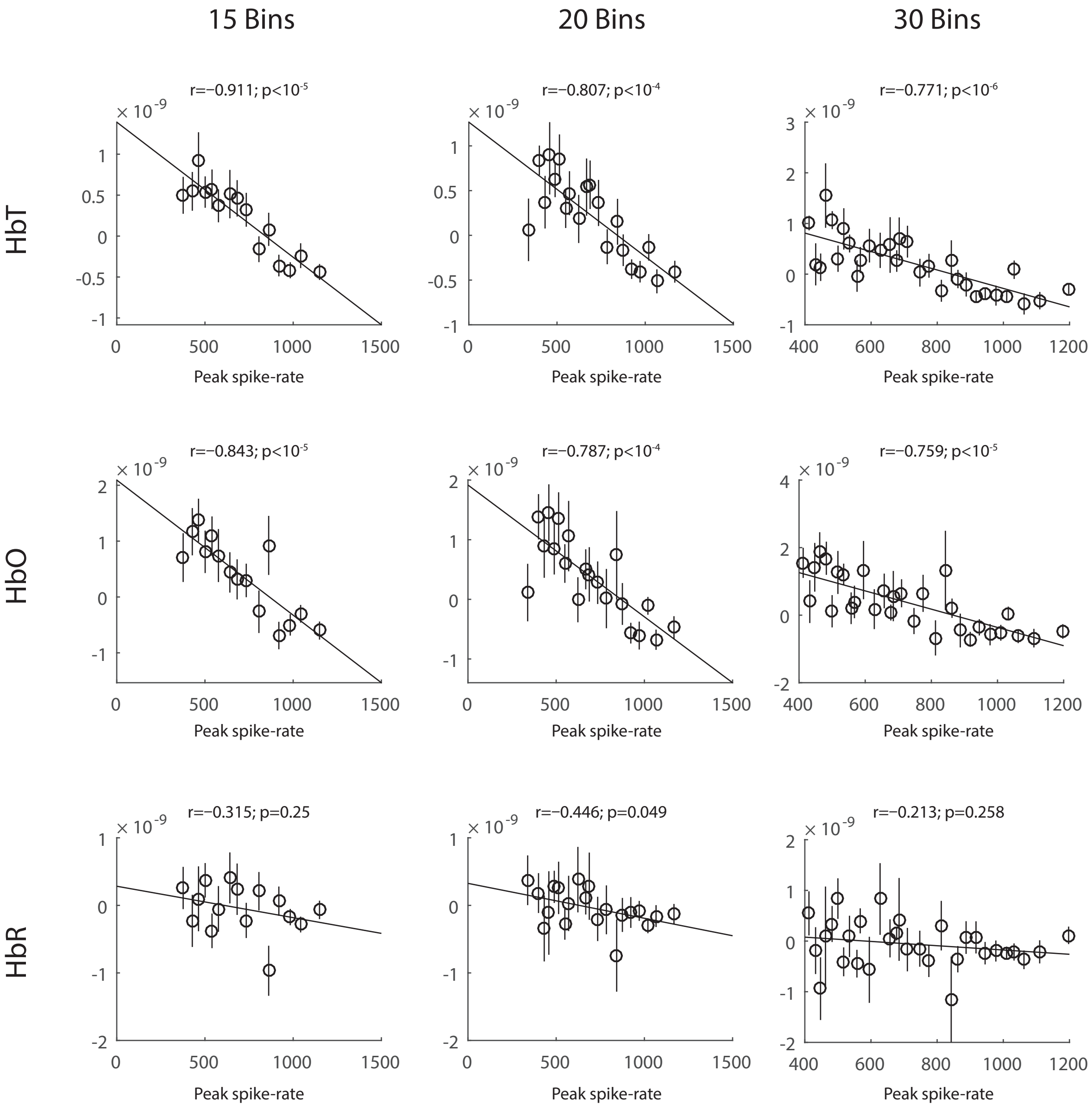

Figure S1. Related to Figure 1. Correlations of spiking activity with HbT, HbO and HbR dips are independent of binsize. Although both HbT and HbO dips showed strong correlations with peaks in spike-rates, the correlations with HbT were stronger than HbO. Although the strength of the correlations got weaker with increasing the number of bins, the significance was not very different. No correlations were observed with the dip strength in HbR. These correlations were independent of the number of groups the data were divided into.

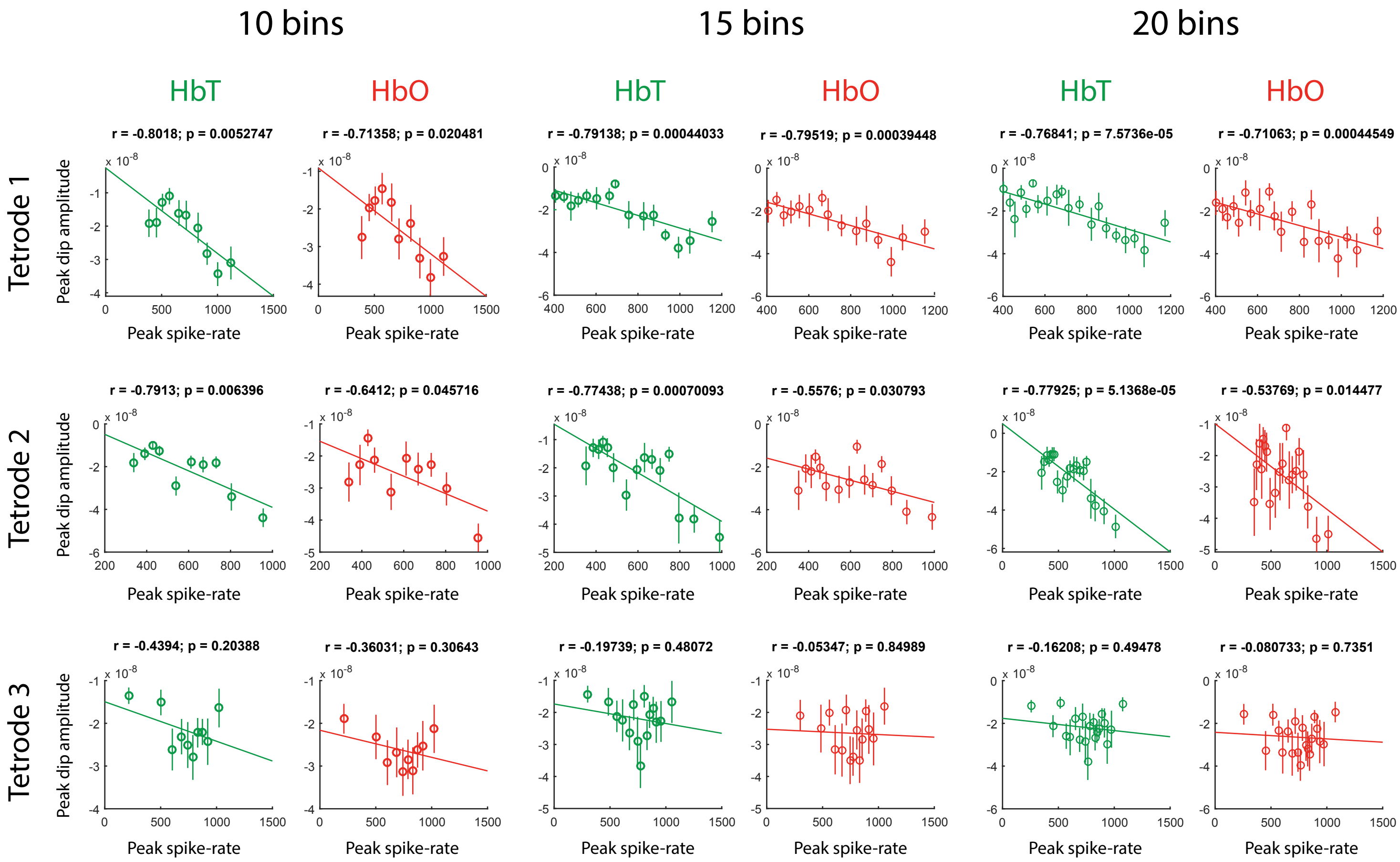

Figure S2. Related to Figure 1. Correlations between peak amplitude of inital dip and peak spike-rates across tetrodes. Instead of using the mean slope between 0-1s, we used the peak dip amplitude (minimum signal value between 0 and 3s) for HbT and HbO and correlated it with the peak spike-rate. We found that the correlations of peak dip amplitude with peak spike-rates were strongest on the tetrode closest to the emitter, and decreased with increasing distance of tetrode from emitter (see figure 2H for comparison). These results were independent of the number of bins the trials were divided into. These results demonstrate that both HbO and HbT dip amplitudes can be used a proxy for underlying spiking activity.

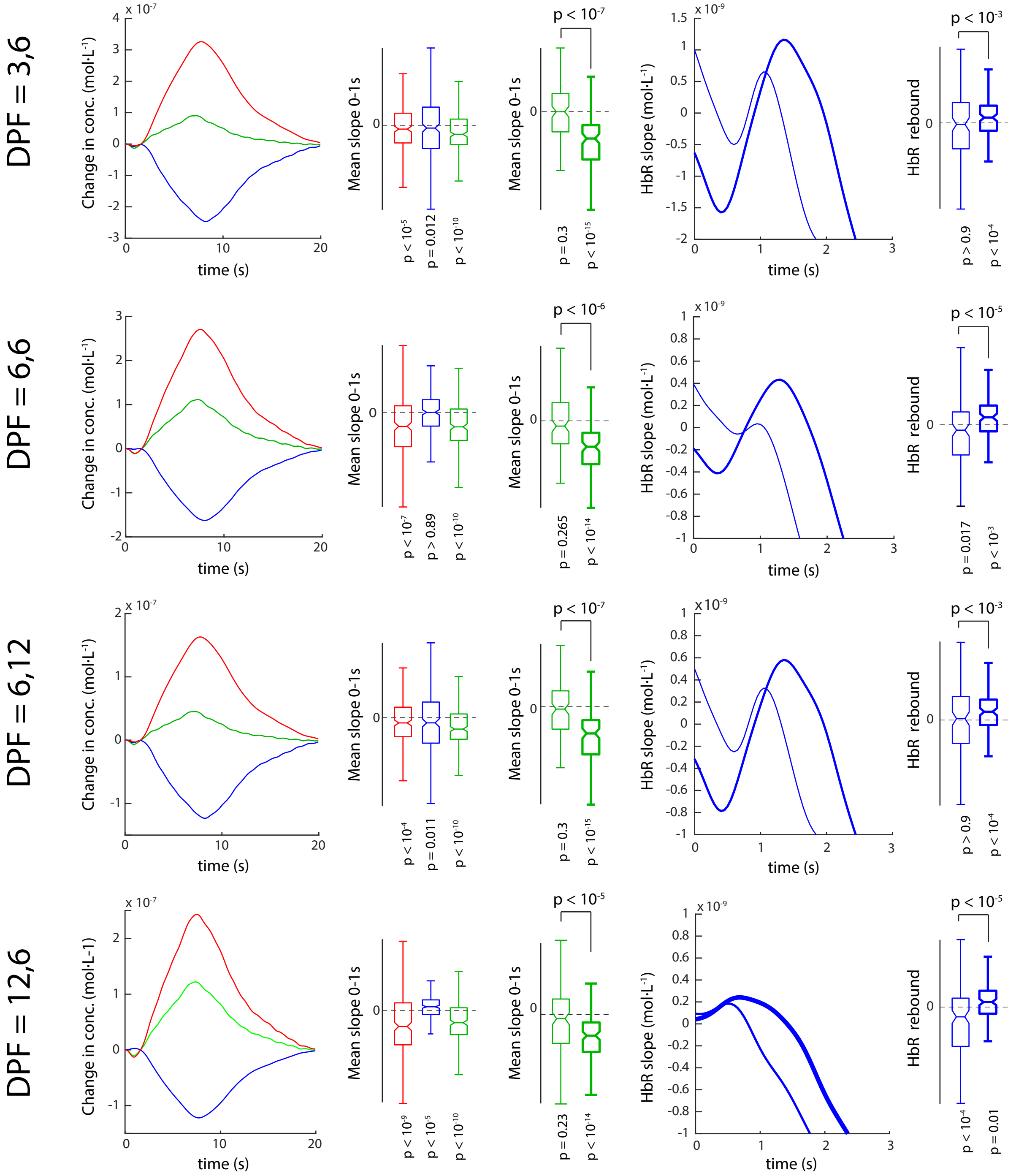

Figure S3. Verification of results across various differential path length factors (DPF). The conversion of optical density to concentration may vary based on the choice of DPF[1]. To ensure that our results did not depend on the DPF, we used multiple combinations of physiologically relevant DPF as reported earlier[1] (the pair of values represent the DPFs at 760 and 850nm resp.) and found that although different DPFs lead to a change in the amplitude of the signals, the overall results were not affected. For each combination of DPFs, we observed strong dips in the HbO and HbT signals. We also observed an increase in the dip strength for HbT during high spiking trials. Low spiking trials failed to elicit a significant dip. The HbR signal also elicited a strong dip and rebound modulation in each case. For each combination of DPFs, the results were nearly identical and individually significant.
Interestingly, the DFP combination 12,6 revealed increases in HbR within the 0-2s of trial onset. However, there was no difference in HbR concentration in high-spiking vs low-spiking trials ($p>0.4$; n=130/group, Wilcoxon rank-sum test). Furthermore, the strongest difference between high and low-spiking was still oberved in the HbT-dip ($p<10^{-5}$) than HbO-dip ($p<10^{-3}$).
This reaffirms previous results[1] that, at least in the primary visual cortex, although the choice of DPF might alter the amplitude of the calculated change in concentrations of HbO, HbR and HbT, it does not alter their relationship to underlying spiking activity. Notches represent 95% confidence intervals.

1. Jasdzewski, G., Strangman, G., Wagner, J., Kwong, K.K., Poldrack, R.A. and Boas, D.A., 2003. Differences in the hemodynamic response to event-related motor and visual paradigms as measured by near-infrared spectroscopy. Neuroimage, 20(1), pp.479-488.

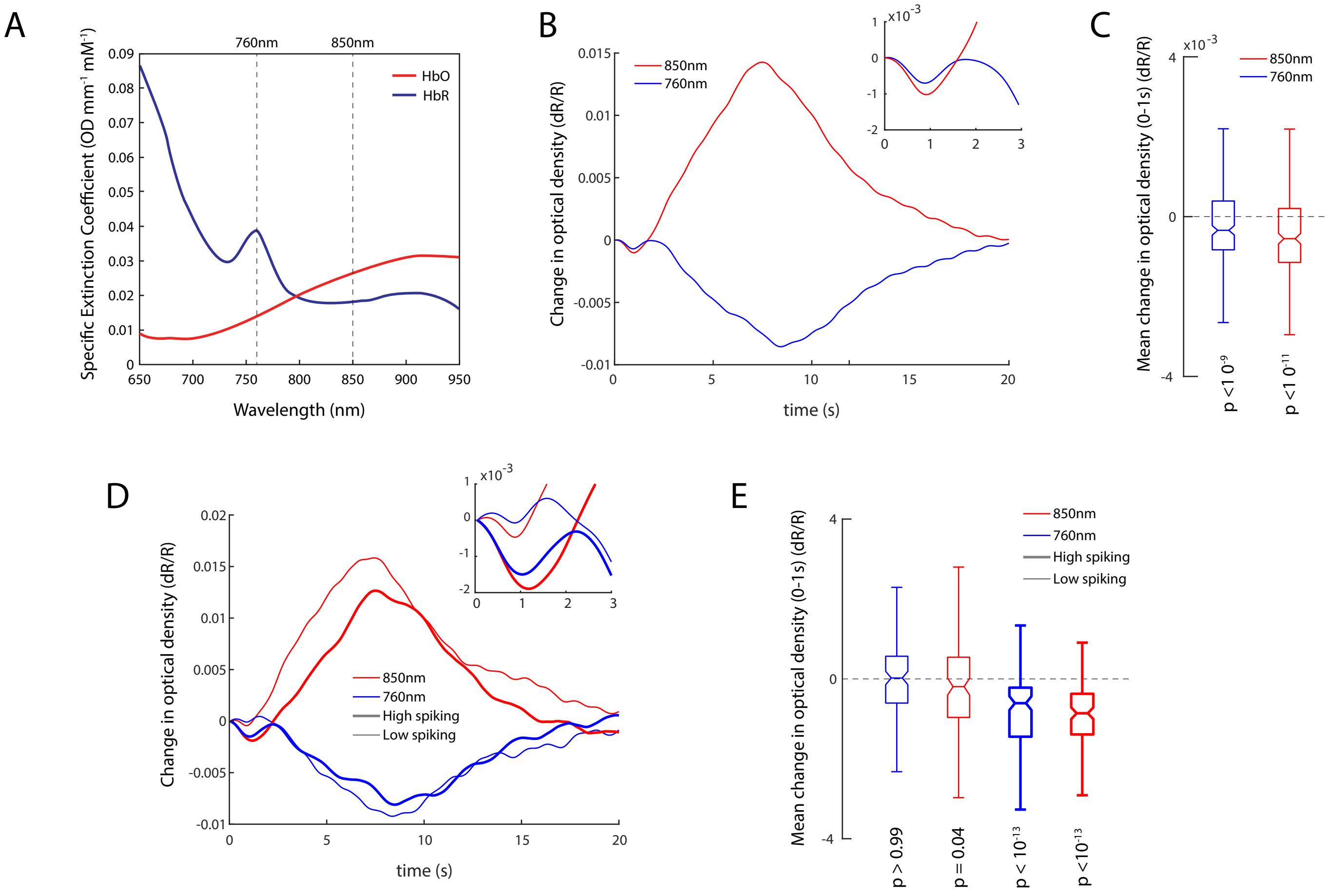

Figure S4. Changes in optical density for both imaging wavelenghts (760nm and 850nm) reveal an early decrease in chromophore concentration. A) Specific extinction coefficients for HbO and HbR as a function of wavelength. Although both HbO and HbR absorb light at both wavelengths, 760nm is more sensitive to HbR changes whereas 850nm is more sensitive to HbO changes. Changes in the observed optical density are proportional to changes in the chromatophores according to Beer's law. B) Mean changes in the optical densities at 760nm and 850nm across all 260 trials. Inset represents the changes between 0-3s. A decrease in optical density for both wavelenghts can be observed. C) Mean change in optical density between 0 and 1s for 760 and 850nm. The distributions are significanlty less than zero. D) Optical density changes for trials with high and low spiking activity. Inset represents the changes between 0-3s. Larger decreases in optical density are observed for the high spiking trials. E) Mean changes in optical density between 0-1s reveal significant decreases in optical density for high-spiking trials. The decrease in optical density for both chromatophores implies a decrease in both HbO and HbR concentrations. Since HbT is the sum of changes in HbO and HbR, simultaneous decreases in HbO and HbR would lead to larger decreases in HbT when changes in optical density are converted to changes in concentration. Surprisingly, we found mildly significant decreases i n the optical density at 850nm for low-spiking trials. However, after conversion to concentration changes, none of the traces (HbO, HbR or HbT) revealed significant changes between 0-1s.

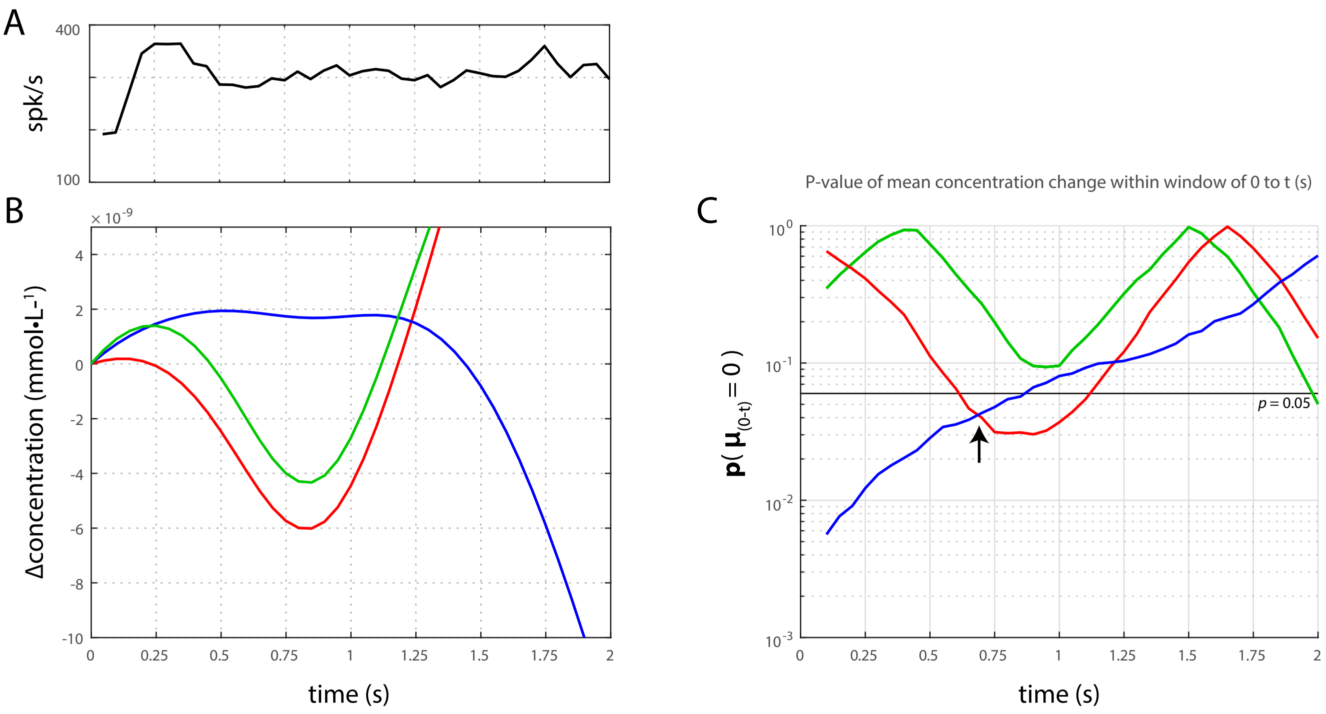

Figure S5. Further analysis of low-spiking trials reveals a significant increase in HbR and decrease in HbO concentrations within the first 750ms. We further analyzed trials where the peak spike-rate was less than the median value of all trials (**A**, see also Figure 2A). Although these trials belonged to the lower half of the distribution, they still had significantly high peak spike-rates and stimulus induced spike-rate moduations (see Fig. 2D). We obtained the mean HbO, HbR and HbT traces from these trials (**B**). Increases in HbR traces and decreases in the HbO traces are clearly observable within these trials. We determined the temporal window within which these changes were significant, by incrementing the width of the window from 0 to 2s (**C**, $p<0.05$ based on the Wilcoxon signed-rank test). We found the changes to be significant for HbO and HbR within 0-0.7s of stimulus onset (arrow). We failed to find significant changes for HbT for the same period (**C**, green trace). These findings demonstrate that during conditions of low spiking, the initial-dip consists of increases in HbR and decreases in HbO, and the HbT dip is absent. This suggets that the HbT dip is only induced during conditions of strong bursts in spiking activity.

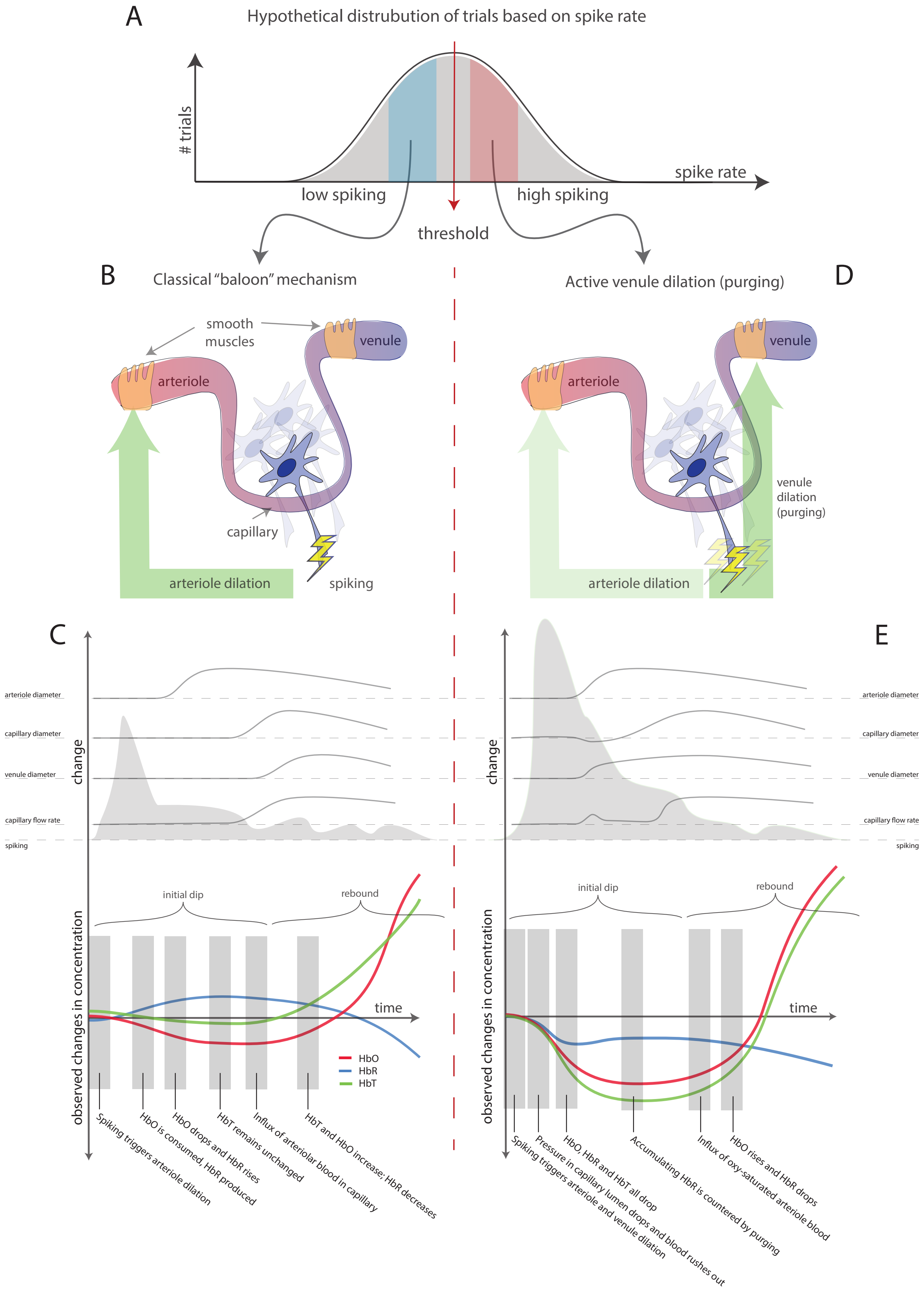

Figure S6. Illustration of proposed mechanism of purging and it's effects on hemodynamic signals.

A) The distribution of spike-rate seems to have a threshold that allows teasing apart the two distinct mechanisms of the initial dip, observed between 0-2s after stimulus onset. **B)** Low spiking activity triggers distal arteriole dilation, and the influx of blood has a short but significant delay, and the ongoing spiking activity leads to a temporary increase of HbR and a decrease in HbO, while no changes in HbT are observed **(C)**. However, high spiking activity induces active venule dilation **(D)**, which causes a sudden drop in capillary pressure, causing blood to rush out. The drop in pressure in the capillary also increases the blood flow through the capillary, draining capillary blood at a faster rate. This process enforces an upper limit on the concentration of HbR. These changes are observed as a sudden drop in HbT, and hence both HbO and HbR **(E)**.

Supplementary table 1. Hemodynamic initial dips during spontaneous and stimulus-induced activity.

dataset	parameter	condition	mean	SEM	p-value*	Sample-size
Stimulus-induced activity	Peak spike-rate (spk/s)	all trials	705.85	14.7	$<10^{-10}$	260
		high-spiking	899.96	12.89	$<10^{-10}$	122
		Low-spiking	497.28	8.35	$<10^{-10}$	125
	HbO dip (μmol·L^{-1})	All trials	-0.52	0.12	$<10^{-7}$	260
		high-spiking	-0.87	0.146	$<10^{-11}$	122
		Low-spiking	-0.22	0.188	0.21	125
	HbR dip (μmol·L^{-1})	All trials	-0.04	0.078	0.89	260
		high-spiking	-0.2	0.089	0.0076	122
		Low-spiking	0.09	0.12	0.18	125
	HbT dip (μmol·L^{-1})	All trials	-0.56	0.11	$<10^{-10}$	260
		High-spiking	-1.08	0.112	$<10^{-14}$	122
		Low-spiking	-0.13	0.179	0.287	125
	HbR buildup (μmol·L^{-1})	All trials	-0.041	0.805	0.634	260
		High-spiking	0.236	0.093	$<10^{-3}$	122
		Low-spiking	-0.365	0.133	0.007	125
	HbR- modulation (epoch II – epoch I)	All trials	0.029	0.095	0.135	260
		High-spiking	0.534	0.077	$<10^{-10}$	122
		Low-spiking	-0.481	0.165	$<10^{-3}$	125
	HbO/HbT dip (%)	High-spiking	50.36	17	-	122
Spontaneous activity	Total spike count	All runs	$1.56\text{x}10^{5}$	$1.03\text{x}10^{4}$	$<10^{-3}$	16
		High-spiking	$1.86\text{x}10^{5}$	$5.81\text{x}10^{3}$	0.0078	8
		Low-spiking	$1.27\text{x}10^{4}$	$1.36\text{x}10^{4}$	0.0078	8
	HbO dip (a.u.)	All runs	-0.034	0.005	$<10^{-6}$	48
		High-spiking	-0.051	0.012	$<10^{-3}$	80
		Low-spiking	-0.036	0.015	0.06	80
	HbR dip (a.u.)	All runs	-0.009	0.005	0.1	48
		High-spiking	-0.032	0.014	0.0041	80
		Low-spiking	-0.001	0.016	0.9	80
	HbT dip (a.u.)	All runs	-0.043	0.007	$<10^{-6}$	48
		High-spiking	-0.07	0.016	$<10^{-5}$	80
		Low-spiking	-0.037	0.018	0.122	80
	HbR buildup (a.u.)	High-spiking	0.04	0.0136	0.011	80
		Low-spiking	0.039	0.012	0.049	80
	HbR- modulation	High-spiking	0.072	0.024	0.0017†	80
		Low-spiking	0.041	0.031	0.16†	80
	HbO/HbT dip (%)	High-spiking	58.9	32	-	48

* Wilcoxon's two-tailed sign-rank test
† Wilcoxon's one-tailed sign-rank test
